# Supplementary material for: Long-Term Outcome of Critically Ill Advanced Cancer Patients Managed in an Intermediate Care Unit
Source: J Clin Med. 2022 Jun 16;11(12):3472. doi: 10.3390/jcm11123472 (PMC9225024; doi:10.3390/jcm11123472)
Supplement: Supplementary file 1 [file jcm-11-03472-s001.zip › jcm-1744277-supplementary.pdf]

Table S1. Univariate analysis of risk factors associated with hospital mortality

Variables	Non survivors	Survivors	P value
Patients, n	90	150	
Male, n (%)	60 (66.6)	99 (66)	0.516
Age	63.6 (12.4)	61.9 (13.6)	0.325
BMI	24.8 (4.9)	24.5 (4.7)	0.607
Diabetes Mellitus, n (%)	21 (23.3)	32 (21.3)	0.418
CHF, n (%)	9 (10)	12 (8)	0.641
Ischemic heart disease, n (%)	9 (10)	8 (5.3)	0.198
COPD, n (%)	11 (12.2)	29 (19.3)	0.210
Hypertension, n (%)	33 (36.7)	52 (34.7)	0.781
Cirrhosis, n (%)	13 (14.4)	5 (3.3)	0.004
Charlson Score, n (%)<br>• > 7	 78 (86.7)	 123 (82)	0.372
ECOG, n (%)<br>• 3-4	 44 (48.9)	 40 (26.7)	0.001
Functional Status, n (%)<br>• Partially dependent/dependent	 83 (92.2)	 128 (85.3)	0.152
On any immunosuppressive treatment *	77 (85.6)	106 (70.7)	0.012
Solid cancer, n (%)	87 (96.7)	142 (94.7)	0.463
DNR orders, n (%)	60 (66.6)	54 (36)	0.000
LOS (days)	23.8 (21.6)	23.7 (31.5)	0.966
LOS ImCU (days)	5.8 (5.2)	4.6 (4)	0.063
Location before ImCU admission, n (%)<br>• Emergency room<br>• ICU<br>• Conventional<br>• Other	 <br>22 (24.4)<br>7 (7.8)<br>56 (65.6)<br>2 (2.2)	 <br>52 (34.7)<br>11 (7.3)<br>84 (56)<br>3 (2)	0.424
Urgent admission to ImCU, n (%)	79 (87.8)	131 (87.3)	0.92
Main cause of admission to ImCU, n (%)<br>• Sepsis<br>• Acute respiratory failure (excluding sepsis)<br>• Post-surgical/Complex patient monitoring<br>• Cardiovascular<br>• Neurologic<br>• Other ((hidroelectrolitic disturbance,bleeding …)	 <br>34 (37.8)<br>36 (40)<br><br>4 (4.4)<br><br>6 (6.7)<br>6 (6.7)<br>4 (4.4)	 <br>43 (28.7)<br>57 (38)<br><br>18 (12)<br><br>13 (8.6)<br>4 (2.7)<br>15 (10)	0.096
Respiratory insufficiency, n (%) **	65 (72.2)	92 (61.3)	0.086
Need for non-invasive mechanical ventilation, n (%)	44 (48.9)	44 (29.3)	0.004
Use of vasoactive drugs, n (%)	14 (15.6)	12 (8)	0.068
Reason for discharge from ImCU, n (%)<br>• Improvement<br>• Worsening	 35 (38.9)<br>28 (31.1)<br>26 (28.9)	 133 (88.7)<br>8 (5.3)<br>0	< 0.001

• *Death*<br>• Other	1 (1.1)	9 (6)	
Hemoglobin (g/dl) ***	9.9 (1.8)	10.1 (1.8)	0.467
Leucocytes (10E9/L) ***	11.8 (8.4)	11.1 (8.8)	0.531
Platelet count (10E9/L) ***	181.2 (153.6)	247 (165)	0.003
Reactive C Protein (mg/dl) (NV < 0.4) ***	14.8 (11.4)	15.8 (11.6)	0.548
Procalcitonin (ng/ml) ***	6.3 (16.1)	8.7 (22.8)	0.406
Creatinine (mg/dl) ***	1.3 (0.9)	1.2 (1.1)	0.756
Urea (mg/dl) ***	0.68 (0.4)	0.50 (0.3)	0.001
Albumin (g/dl)***	2.3 (0.6)	2.4 (0.6)	0.681
Bilirubin (mg/dl) ***	3.1 (5.2)	1.7 (2.9)	0.030
SAPS 3 (number)	73 (13)	65 (12)	< 0.001
SAPS 3 (probability, %)	59.8 (19.6)	47.3 (20.2)	< 0.001

All values expressed as mean (d.s), otherwise specified. * Steroids, chemotherapy or other immunosuppressive treatment – e.g Rituximab, methotrexate…. – six months prior to admission; ** patients with SpO2 < 90 % regardless of the main cause of admission (e.g pleural effusion, pneumonia, fluid overload, pulmonary embolism …); *** at ImCU admission. BMI: Body mass index; CHF: chronic heart failure; COPD: Chronic obstructive pulmonary disease; ECOG: Eastern Cooperative Oncology Group; DNR: Do not resuscitate; LOS: Length of stay; ImCU: Intermediate care unit; ICU: Intensive care unit; NV: Normal value; SAPS: Simplified Acute Physiology Score.

Table S2. Univariate analysis of risk factors associated with 30 days Mortality

Variables	Non survivors	Survivors	P value
Patients, n	125	115	
Male, n (%)	80 (64)	79 (68.7)	0.442
Age	62.9 (12.7)	62 (13.6)	0.574
BMI	24.9 (4.8)	24.2 (4.7)	0.187
Diabetes Mellitus, n (%)	28 (22.4)	25 (21.7)	0.902
CHF, n (%)	12 (9.6)	9 (7.8)	0.627
Ischemic heart disease, n (%)	10 (8)	7 (6.1)	0.564
COPD, n (%)	17 (13.6)	23 (20)	0.184
Hypertension, n (%)	42 (33.6)	43 (37.4)	0.540
Cirrhosis, n (%)	15 (12)	3 (2.6)	0.006
Charlson Score, n (%)<br>• 0-6<br>• > 7	 17 (13.6)<br>108 (86.4)	 22 (19.1)<br>93 (80.9)	0.246
ECOG, n (%)<br>• 0-2<br>• 3-4	 71 (56.8)<br>54 (43.2)	 85 (73.9)<br>30 (26.1)	0.005
Functional Status, n (%)<br>• Independent<br>• Partially dependent/Dependent	 11 (8.8)<br>114 (91.2)	 18 (15.7)<br>97 (84.3)	0.104
On any immunosuppressive treatment *	100 (80)	83 (72.2)	0.155
Solid cancer, n (%)	121 (96.8)	108 (93.9)	0.285
DNR orders, n (%)	79 (63.2)	35 (30.4)	0.000
LOS (days)	23.7 (21.6)	23.8 (33.9)	0.977
LOS ImCU (days)	5.8 (5)	4.3 (3.7)	0.008
Location before ImCU admission, n (%)<br>• Emergency room<br>• ICU<br>• Conventional<br>• Other	 34 (27.2)<br>10 (8)<br>79(63.2)<br>2 (1.6)	 40 (34.7)<br>8 (7)<br>64 (55.7)<br>3 (2.6)	0.558
Urgent admission to ImCU, n (%)	110 (88)	100 (87)	0.807
Main cause of admission to ImCU, n (%)<br>• Sepsis<br>• Acute respiratory failure (excluding sepsis)<br>• Post-surgical/Complex patient monitoring<br>• Cardiovascular<br>• Neurologic<br>• Other ((hidroelectrolitic disturbance,bleeding …)	 42 (33.6)<br>52 (41.6)<br>8 (6.4)<br>9 (7.2)<br>6 (4.8)<br>8 (6.4)	 35 (30.4)<br>41 (35.6)<br>14 (12.2)<br>10 (8.7)<br>4 (3.5)<br>11 (9.6)	0.648
Respiratory insufficiency, n (%) **	88 (70.4)	69 (60)	0.091
Need for non-invasive mechanical ventilation, n (%)	56 (44.8)	32 (27.8)	0.006
Use of vasoactive drugs, n (%)	18 (14.4)	8 (7)	0.064
Reason for discharge from ImCU<br>• Improvement<br>• Worsening<br>• *Death*<br>• Other	 60 (48)<br>31 (24.8)<br>26 (20.8)<br>8 (6.4)	 108 (93.9)<br>5 (4.4)<br>-<br>2 (1.7)	< 0.001

Hemoglobin (g/dl) ***	9.9 (1.9)	10.2 (1.8)	0.240
Leucocytes (10E9/L) ***	12.5 (8.6)	10.1 (8.5)	0.031
Platelet count (10E9/L) ***	204.2 (157.7)	241.4 (168.4)	0.080
Reactive C Protein (mg/dl) (NV < 0.4) ***	16.3 (12.5)	14.4 (10.3)	0.193
Procalcitonin (ng/ml) ***	8.8 (23.1)	6.7 (17.2)	0.477
Creatinine (mg/dl) ***	1.3 (0.9)	1.2 (1.1)	0.878
Urea (mg/dl) ***	0.64 (0.43)	0.48 (0.35)	0.002
Bilirubin (mg/dl) ***	2.8 (4.7)	1.6 (2.8)	0.023
Albumin (g/dl)***	2.3 (0.6)	2.4 (0.6)	0.668

All values expressed as mean (d.s), otherwise specified. * Steroids, chemotherapy or other immunosuppressive treatment – e.g Rituximab, methotrexate…. – six months prior to admission; ** patients with SpO2 < 90 % regardless of the main cause of admission (e.g pleural effusion, pneumonia, fluid overload, pulmonary embolism …); *** at ImCU admission. BMI: Body mass index; CHF: chronic heart failure; COPD: Chronic obstructive pulmonary disease; ECOG: Eastern Cooperative Oncology Group; DNR: Do not resuscitate; LOS: Length of stay; ImCU: Intermediate care unit; ICU: Intensive care unit; NV: Normal value.
